# Polysaccharide-Stabilized Selenium Nanoparticles Derived from *Phellinus igniarius* Regulate Growth and Metabolic Reprogramming in Rice

**DOI:** 10.3390/plants15040632

**Published:** 2026-02-16

**Authors:** Qingpan Bu, Ping Li, Haiyuyan Yang, Xiaodan Wang, Yinghui Gu, Lihui Zhang, Kai Song

**Affiliations:** 1 School of Life Science, Changchun Normal University, Changchun 130032, China; buqingpan@ccsfu.edu.cn (Q.B.); chunlps@163.com (P.L.); yinxiaotangyhyy@sina.com (H.Y.); wangxdann@163.com (X.W.); guyinghui0118@163.com (Y.G.); 2 Institute of Innovation Science and Technology, Changchun Normal University, Changchun 130032, China

**Keywords:** selenium nanoparticles, *Phellinus igniarius* polysaccharides, rice, antioxidant regulation, metabolomic reprogramming, nano-agriculture

## Abstract

To address the instability of conventional selenium fertilizers, we developed *Phellinus igniarius* polysaccharide-stabilized selenium nanoparticles (SH-SeNPs). These ~90 nm nanoparticles exhibited excellent stability and enhanced antioxidant capacity compared with native polysaccharides. Foliar application significantly promoted the early growth and biomass of rice without inducing oxidative stress. Specifically, treatment with 5 mg/L SH-SeNPs increased the root length from 5.22 ± 0.78 cm (control) to 5.91 ± 0.50 cm, while the 45 mg/L treatment increased the shoot length from 1.63 ± 0.27 to 1.89 ± 0.35 cm during germination. Mechanistically, SH-SeNPs maintained redox homeostasis through selective enzymatic regulation. Metabolomic analysis indicated a potential strategic metabolic reprogramming: SH-SeNPs appeared to induce the diversion of carbon–nitrogen flux from free amino acids toward the shikimate and phenylpropanoid pathways. This proposed “efficient defense–robust growth” balance suggests that SH-SeNPs may act not merely as a nutrient source but also as a metabolic regulator. These findings provide insights into the mechanisms through which polysaccharide-stabilized SeNPs regulate growth and metabolism in rice during early growth stages, highlighting their potential as nano-biostimulants for seedling establishment.

## 1. Introduction

Although selenium (Se) is an essential micronutrient for human health, widespread soil Se deficiency limits its dietary intake globally [1,2,3,4,5,6,7]. Current agronomic biofortification strategies using inorganic Se fertilizers are effective but often constrained by narrow safety margins and potential environmental toxicity [8,9]. Selenium nanoparticles (SeNPs) have emerged as a promising alternative due to their lower toxicity and high bioavailability. However, bare SeNPs are thermodynamically unstable and prone to aggregation, severely limiting their practical application in agriculture [10,11,12]. While surface functionalization with biopolymers offers a solution to improve their stability, the selection of functionalizing agent is critical for maximizing biological efficacy.

Despite the fact that polysaccharide-stabilized SeNPs have demonstrated synergistic bioactivities in biomedical contexts [13,14,15], their application in crop systems remains largely underexplored. Previous research has typically regarded polysaccharides merely as inert stabilizers, overlooking their potential as active metabolic regulators in plants. Consequently, a critical knowledge gap remains regarding how functionalized SeNPs interact with plant metabolic networks.

*Phellinus igniarius* (*P. igniarius*)—a medicinal fungus renowned for its potent antioxidant and immunomodulatory polysaccharides [16,17,18]—represents a novel source of biologically active stabilizers for nano-agriculture [19,20,21]. Unlike common structural polysaccharides, *P. igniarius* polysaccharides (SH) may confer specific stress resistance traits to crops. To date, the physiological impacts and metabolic regulatory mechanisms of polysaccharide-stabilized SeNPs (SH-SeNPs) in rice, particularly regarding carbon–nitrogen reallocation, have not been systematically elucidated.

Therefore, this study aims to bridge this gap by synthesizing SH-SeNPs and evaluating their efficacy in rice biofortification. We hypothesize that SH-SeNPs act not only as a nutrient source but also as a metabolic primer, inducing strategic reprogramming of the carbon–nitrogen flux toward defense-related secondary metabolism. By integrating material characterization, physiological assessment, and untargeted metabolomics, this work seeks to explore the potential “efficient defense–robust growth” mechanism enabled by this novel fungal-derived nanocomposite.

## 2. Results

### 2.1. Synthesis and Characterization of SH-SeNPs

**Figure 1:**
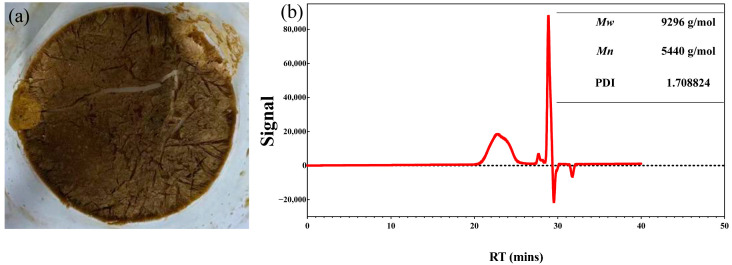
Physicochemical characterization of *P. igniarius* polysaccharides. (**a**) Appearance of the freeze-dried crude polysaccharide extracted from *P. igniarius*. (**b**) Molecular weight distribution of *P. igniarius* polysaccharides determined by gel permeation chromatography (GPC).

The physicochemical characterization confirmed the successful construction of SH-SeNPs. The extracted *P. igniarius* polysaccharides exhibited a relatively high carbohydrate content and a heterogeneous molecular weight distribution, with a number average molecular weight of 5.44 kDa (Figure 1a,b).

**Figure 2:**
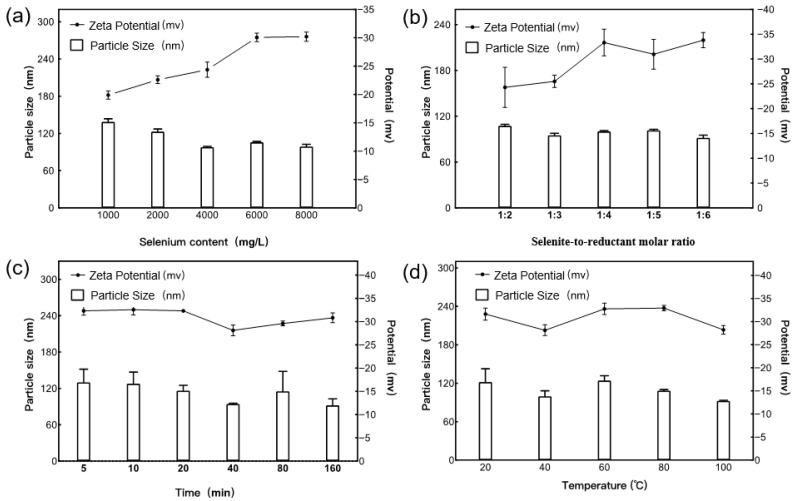
Effects of single-factor conditions on the formation of SH-SeNPs: (**a**) selenium concentration; (**b**) molar ratio of sodium selenite to ascorbic acid; (**c**) reaction time; (**d**) reaction temperature.

Systematic optimization of synthesis parameters revealed that the selenium concentration, reductant ratio, and reaction temperature jointly governed the size and dispersion of the nanoparticles (Figure 2a–d). Response surface modeling demonstrated a highly significant regression (*p* < 0.01; *R^2^* = 0.9941; Table 1 and Table 2), with selenium concentration exerting the strongest influence on particle size. Under optimized conditions, the SH-SeNPs exhibited an average diameter of 91.58 nm, closely matching the model prediction (Table 1).

**Figure 3:**
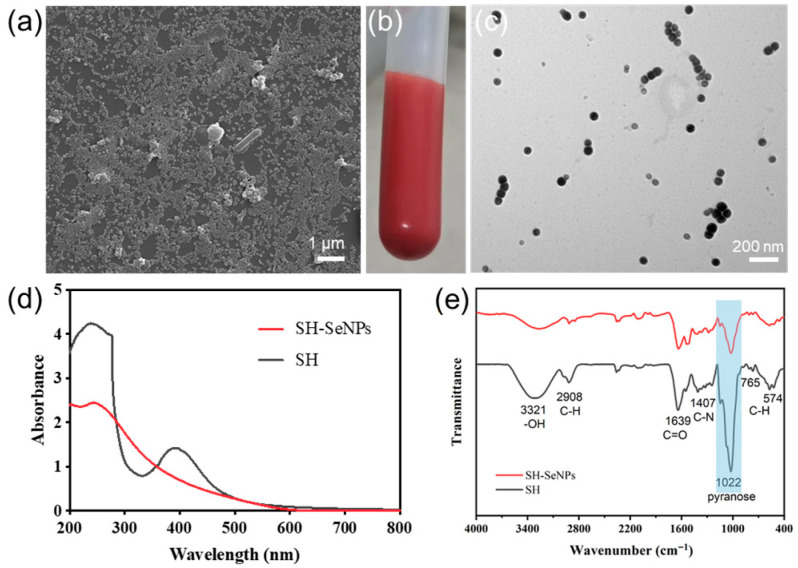
Characterization of the synthesized nanoparticles. (**a**) SEM image of SH-SeNPs (scale bar = 1 μm). (**b**) Photograph of the SH-SeNPs aqueous suspension showing a homogeneous bright-red appearance. (**c**) TEM image of SH-SeNPs with predominantly spherical morphology (scale bar = 200 nm). (**d**) UV–visible absorption spectra of SH and SH-SeNPs. (**e**) FT-IR spectra of SH and SH-SeNPs.

Scanning electron microscopy (SEM) and transmission electron microscopy (TEM) revealed that the SH-SeNPs were predominantly spherical, well dispersed, and exhibited a relatively uniform size distribution, with particle diameters mainly around 90 nm (Figure 3a–c). The homogeneous bright-red appearance and good stability of the SH-SeNPs suspension further indicated effective colloidal stabilization (Figure 3b). UV–visible spectroscopy revealed a broad absorption band in the range of 200–600 nm for SH-SeNPs, which was absent for the non-selenized polysaccharide (Figure 3d). FT-IR spectra displayed characteristic polysaccharide absorption bands, with a notable decrease in the pyranose-ring-associated signal intensity in SH-SeNPs (Figure 3e).

**Figure 4:**
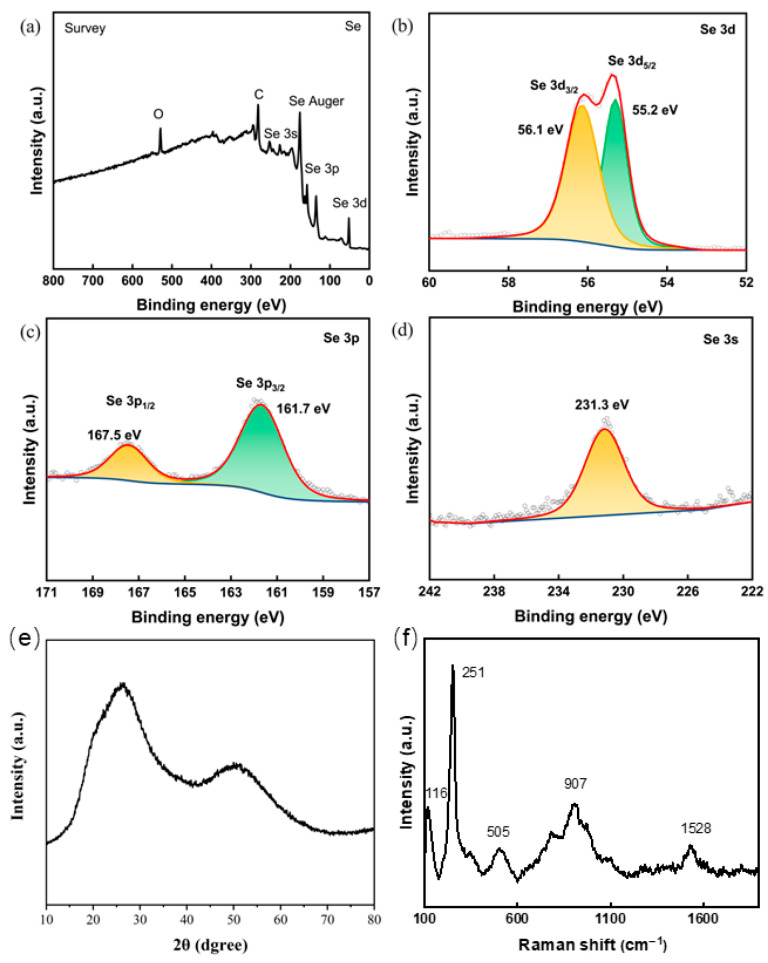
Crystalline structure and selenium valence state analysis of SH-SeNPs: (**a**) XPS survey spectrum of SH-SeNPs; (**b**) Se 3d high-resolution XPS spectrum; (**c**) Se 3p high-resolution XPS spectrum; (**d**) Se 3s high-resolution XPS spectrum; (**e**) X-ray diffraction (XRD) pattern of SH-SeNPs; (**f**) Raman spectrum of SH-SeNPs.

XPS analysis demonstrated that the SH-SeNPs consisted primarily of C, O, and Se, with high-resolution Se 3d spectra confirming selenium predominantly in the zero-valent state [Se (0)] (Figure 4a–d) [22]. The absence of sharp diffraction peaks in the XRD patterns indicated an amorphous structure, which was further supported by the characteristic Se-Se vibrational bands observed in Raman spectra (Figure 4e,f) [23,24].

### 2.2. In Vitro Antioxidant Activity and in Vivo Physiological Response

**Figure 5:**
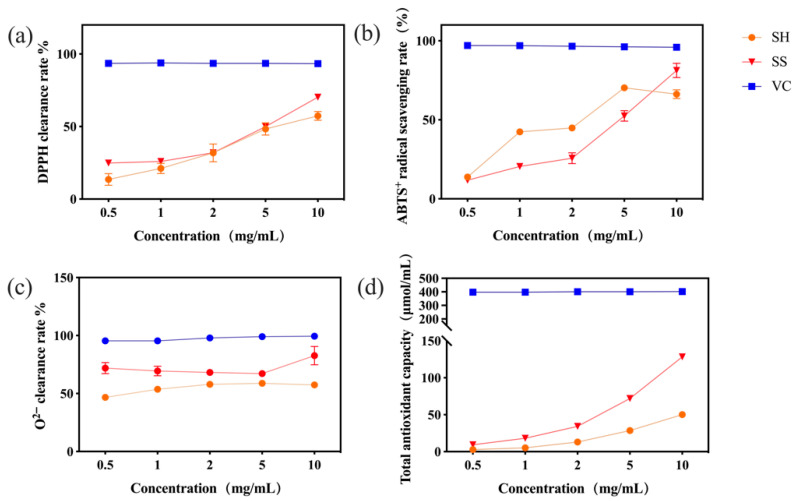
Antioxidant activities of SH and SH-SeNPs: (**a**) DPPH radical scavenging activity; (**b**) ABTS radical scavenging activity; (**c**) superoxide anion (O_2_^−^) scavenging activity; (**d**) total antioxidant capacity, determined via FRAP assay. Abbreviations: SH, *P. igniarius* polysaccharides; SS, SH-SeNPs; VC, Vitamin C (ascorbic acid), used as a positive control. Data are presented as mean ± SD (n = 3).

The SH-SeNPs exhibited significantly higher scavenging activities against DPPH, ABTS^+^, and superoxide anion radicals compared with the non-selenized polysaccharide across the tested concentration range (Figure 5a–c). In parallel, the ferric reducing antioxidant power (FRAP) increased substantially, reaching 128.30 μmol/mL at 10 mg/mL (Figure 5d).

**Figure 6:**
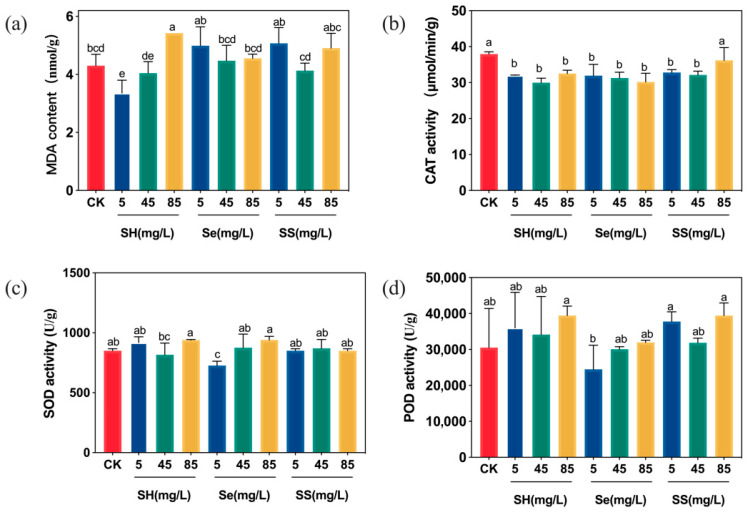
Lipid peroxidation level and antioxidant enzyme activities in rice leaves: (**a**) (MDA) content; (**b**) CAT activity; (**c**) SOD activity; (**d**) POD activity. Treatments: CK, control (deionized water); SH, *P. igniarius* polysaccharides; Se, bare SeNPs; SS, SH-SeNPs. Numbers (5, 45, 85) indicate the concentration of the respective treatment (in mg/L). Different lowercase letters indicate significant differences between treatments (*p* < 0.05).

In rice leaves, foliar application of SH-SeNPs did not increase malondialdehyde (MDA) levels at any tested concentration (Figure 6a). In contrast, high concentrations of the polysaccharide alone significantly increased MDA accumulation. Regarding antioxidant enzymes, neither superoxide dismutase (SOD) nor catalase (CAT) activities differed significantly from control levels across SH-SeNPs treatments (Figure 6b,c). However, peroxidase (POD) activity increased significantly at low (5 mg/L) and high (85 mg/L) SH-SeNP concentrations (Figure 6d).

### 2.3. Effects on Rice Germination, Growth, and Biomass

**Figure 7:**
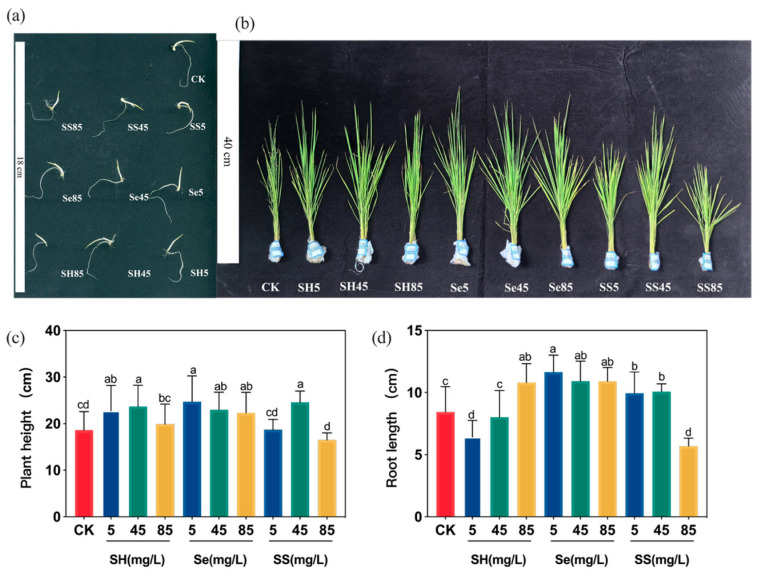
Effects of different selenium treatments on the growth and physiological attributes of rice seedlings: (**a**) phenotypes of rice seedlings after 21 days of cultivation; (**b**) shoot height; (**c**) fresh weight; (**d**) chlorophyll content. Treatments included foliar application of water, polysaccharides, bare SeNPs, and SH-SeNPs. Abbreviations: CK, control; SH, *P. igniarius* polysaccharides; Se, bare SeNPs; SS, SH-SeNPs. Numeric suffixes (5, 45, 85) represent concentrations (in mg/L). Different lowercase letters indicate significant differences among treatments (*p* < 0.05).

Seed germination assays demonstrated that treatment with 45 mg/L SH-SeNPs significantly enhanced both shoot and root elongation compared with the control (Figure 7a; Table 3). During the seedling stage, foliar application of SH-SeNPs significantly increased plant height and root length at moderate concentrations (Figure 7b–d).

**Figure 8:**
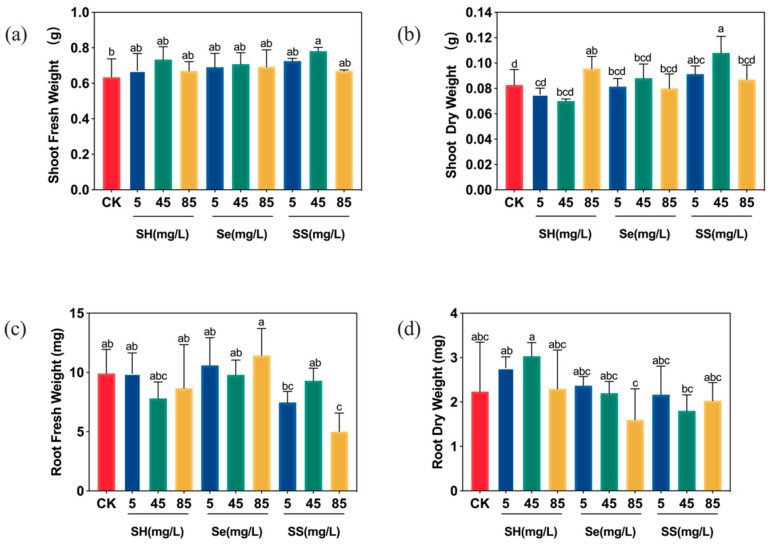
Biomass accumulation of rice seedlings: (**a**) fresh weight of rice shoots; (**b**) dry weight of rice shoots; (**c**) fresh weight of rice roots; (**d**) dry weight of rice roots. Treatments: CK (control), SH (polysaccharides), Se (bare SeNPs), and SS (SH-SeNPs) at concentrations of 5, 45, and 85 mg/L. Different lowercase letters indicate significant differences among treatments (*p* < 0.05).

Biomass analysis showed that low-dose SH-SeNPs significantly increased the shoot dry weight compared with the control, whereas the fresh weight showed no pronounced differences (Figure 8a,b). In roots, high concentrations of SH-SeNPs slightly reduced fresh biomass, while the root dry weight remained comparable to that of the control (Figure 8c,d). The analysis of selenium content in dry tissues indicated that the total selenium content in the shoot dry matter of the 45 mg/L SH-SeNPs group reached 18.44 mg/kg.

### 2.4. Metabolic Reprogramming Underpins Growth Promotion Without Stress

**Figure 9:**
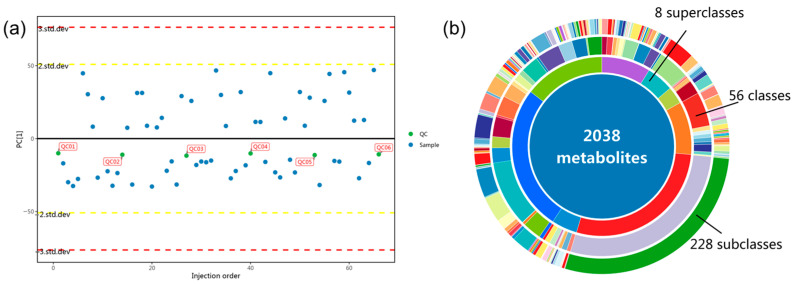
Overview of metabolomic characteristics in rice leaves: (**a**) principal component analysis (PCA) score plot for Control (CK) and 5 mg/L SH-SeNPs (SS5) samples; (**b**) donut chart showing the classification of detected metabolites.

Principal component analysis revealed clear separation between control and low-dose SH-SeNPs (SS5) samples, while tight clustering within groups was observed (Figure 9a). A total of 2038 metabolites spanning eight superclasses were identified (Figure 9b).

**Figure 10:**
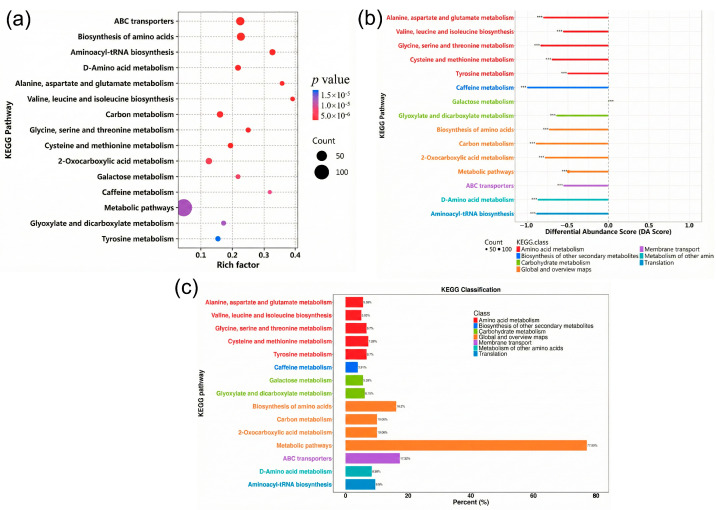
KEGG pathway analysis of differential metabolites between CK and SS5 treatments: (**a**) KEGG enrichment bubble plot; (**b**) KEGG pathway classification. Asterisks “***” indicate significant differences (*p* < 0.001) compared with CK.; (**c**) differential abundance (DA) score analysis of KEGG pathways.

The differential metabolite analysis indicated significant changes in metabolites related to amino acid metabolism, carbon metabolism, and transport processes. Several free amino acids—including serine, asparagine, and tyrosine—were significantly reduced, whereas jasmonic acid and other signaling-related metabolites were increased. KEGG enrichment analysis indicated a significant overrepresentation of amino acid metabolism pathways and ABC transporter-related pathways (Figure 10a,b). The KEGG differential abundance scores further revealed an overall negative shift in free amino acid-associated pathways (Figure 10c).

### 2.5. Activation of Shikimate–Phenylpropanoid Pathways Reflects Strategic Resource Allocation

**Figure 11:**
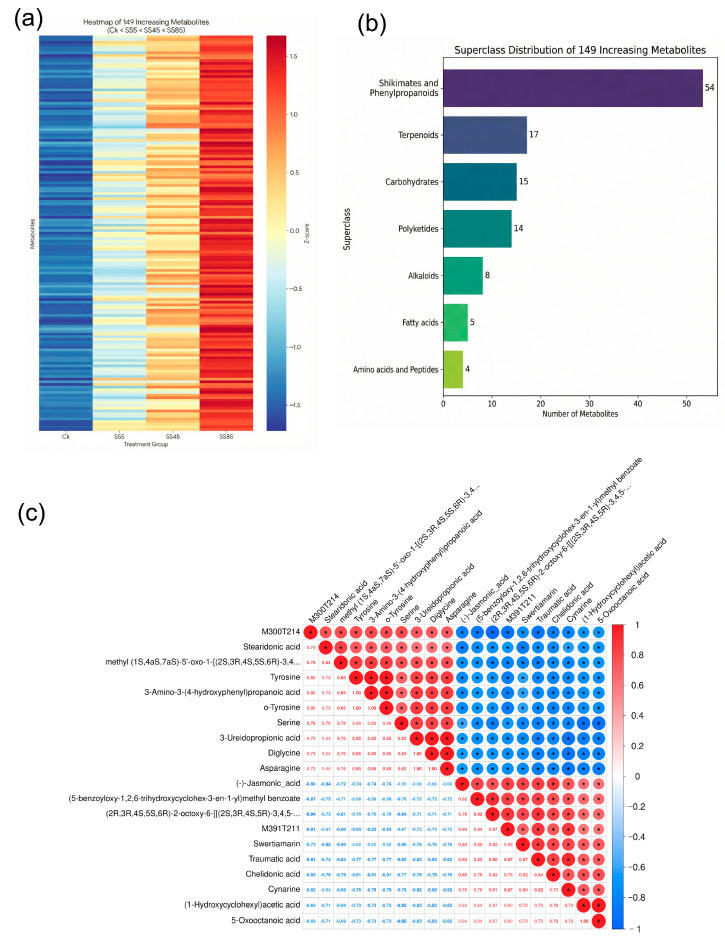
Dose-dependent metabolic responses and correlation analysis induced by SH-SeNPs in rice: (**a**) heatmap showing stepwise accumulation of 149 differential metabolites with increasing SH-SeNPs (SS) concentrations (CK: control; SS5, SS45, SS85: SH-SeNPs at 5, 45, and 85 mg/L); (**b**) superclass composition of metabolites exhibiting dose-dependent responses; (**c**) correlation heatmap between key differential metabolites. An asterisk (*) indicates a statistically significant difference (*P* < 0.05).

Dose–response analysis identified 149 metabolites exhibiting monotonic accumulation with increasing SH-SeNPs concentration (CK < SS5 < SS45 < SS85; Figure 11a). Among these, shikimate- and phenylpropanoid-related metabolites constituted the dominant responsive group, accounting for 36.2% of the total (Figure 11b). Correlation analysis revealed strong negative associations between defense-related secondary metabolites and free amino acids (r < −0.7; Figure 11c).

## 3. Discussion

### 3.1. Stabilization Mechanisms and Physicochemical Properties

The characterization results indicated that *P. igniarius* polysaccharides provided a suitable macromolecular framework for nanoparticle stabilization. The robust process controllability observed in the response surface modeling aligns with previous studies on polysaccharide-mediated synthesis [25]. The FT-IR results suggested the involvement of sugar-ring-related functional groups in nanoparticle stabilization [26]. Collectively, the successful formation of stable, amorphous SH-SeNPs provides a suitable physicochemical basis for subsequent plant uptake and biological investigations [27,28,29].

### 3.2. Redox Homeostasis and Enzymatic Regulation

The stabilized nanostructure translated into a marked enhancement of antioxidant capacity, consistent with reports demonstrating that polysaccharide-functionalized selenium nanoparticles exhibit synergistically enhanced antioxidant activity [30]. Importantly, this elevated antioxidant potential did not result in detectable oxidative damage in plants, whereas the polysaccharide alone at high concentrations did, highlighting that biological activity thresholds remain relevant even for natural macromolecules [31]. The composite architecture of SH-SeNPs appears to buffer the potential adverse effects of individual components.

The selective enzymatic response, particularly the modulation of POD rather than a generalized stress response (SOD/CAT), indicates that SH-SeNPs modulated specific downstream ROS-scavenging processes. Given that POD is involved in both hydrogen peroxide detoxification and cell wall remodeling, such modulation may support growth-related processes while avoiding pronounced oxidative damage [32]. Unlike previous studies where SeNPs typically triggered a generalized upregulation of the entire antioxidant system (SOD, CAT, and POD) to mitigate stress, such as the broad enhancement observed in arsenic-stressed rice [33] or the comprehensive enzyme activation reported in SeNP-biofortified rice [34], our SH-SeNPs induced a more specific response. The selective enhancement of POD, with unchanged SOD and CAT activities, suggests that SH-SeNPs may function more as a mild metabolic modulator rather than a strong stress elicitor. This aligns with the observation by Huang et al. [35], who noted that bio-nano Se fertilizers could improve physiological traits without causing the oxidative burden often associated with inorganic selenium forms.

### 3.3. Growth Promotion Characteristics

SH-SeNPs promoted germination and early seedling development in a concentration-dependent manner [36]. The biomass results indicated a typical biphasic response consistent with micronutrient-mediated regulation [37]. Moderate concentrations (45 mg/L) exerted the optimal growth-promoting effect, while the highest concentration (85 mg/L) induced a decline in growth parameters, indicating a potential inhibitory threshold. These findings confirm that SH-SeNPs effectively enhance early vegetative growth and seedling establishment. The growth-promoting efficacy observed here may also be attributed to the specific particle size (~90 nm) of SH-SeNPs. Cheng et al. recently demonstrated that larger SeNPs (110 nm) significantly enhanced rice biomass and Se bioavailability compared with smaller counterparts (30 nm) [38], which were prone to inducing excessive iron plaque formation on roots. Our SH-SeNPs, with a diameter close to this optimal range, likely achieved a balance between cellular uptake and reduced root surface adsorption, thereby maximizing growth benefits similar to the yield improvements reported in other bio-nano Se applications [35].

### 3.4. Metabolic Reprogramming and Resource Allocation

The metabolomic data point to potential alterations in nitrogen-related metabolism and transmembrane transport processes associated with selenium redistribution [39]. The observed reduction in free amino acid pools does not necessarily indicate nitrogen deficiency but may reflect changes in nitrogen utilization patterns, which may be associated with a redistribution of nitrogen from free intermediates toward other metabolic processes [40,41].

The dose-dependent accumulation of shikimate- and phenylpropanoid-related metabolites (such as styrylpyrone and flavonoid derivatives) suggests a coordinated adjustment favoring secondary metabolism. This pattern aligns with the “efficient defense-robust growth” balance, where resources are strategically allocated toward defense-related secondary metabolism [42,43,44,45]. This metabolic shift mirrors findings by Chen et al., who observed that Se nanomaterials promoted flavonoid biosynthesis and activated defense pathways (SA/JA signaling) in rice [46]. Furthermore, the observed reduction in free amino acids is consistent with Xiong et al., who reported that while low Se concentrations enhance amino acid synthesis, higher or specific thresholds can inhibit it or redirect nitrogen flux [47]. Our results imply that SH-SeNPs may drive this flux towards shikimate-phenylpropanoid pathways, potentially serving as a priming mechanism similar to the “efficient defense” strategy described in recent metabolomic studies of Se-treated crops [48]. It should be noted that these inferences are based on metabolite correlations and pathway enrichment analysis and have not yet been validated at the molecular or enzymatic level.

### 3.5. Mechanism of Uptake and Implications for Nano-Agriculture

Considering the average diameter of ~91.58 nm, which typically exceeds the size limit of cell wall pores (5–20 nm), we hypothesize that SH-SeNPs may enter plant cells predominantly through endocytosis-like mechanisms rather than passive diffusion. While direct cytological evidence is required to confirm this pathway, previous studies on similar-sized nanoparticles suggest that such endocytic entry could allow the polysaccharide-coated selenium to bypass the cell wall barrier, potentially facilitating its subsequent metabolic integration [49].

From an agronomic perspective, the ability of SH-SeNPs to support early seedling growth without inducing metabolic disturbances aligns with the principles of sustainable precision agriculture [50]. However, as this study focused on the seedling stage, long-term field experiments will be required to validate these findings for yield improvement and grain biofortification.

## 4. Conclusions

In this study, we successfully developed a green synthesis strategy for functionalized selenium nanoparticles using *P. igniarius* polysaccharides. The resulting SH-SeNPs (~90 nm) demonstrated superior colloidal stability and amorphous structural characteristics, effectively overcoming the aggregation issues often associated with bare SeNPs.

Mechanistically, the bioactivity of SH-SeNPs in rice seedlings was driven by a distinct physiological regulation strategy rather than a generalized stress response. Unlike inorganic selenium forms that often trigger broad antioxidant system activation, SH-SeNPs promoted seedling growth by selectively upregulating POD activity while maintaining SOD and CAT at basal levels. This selective enzymatic modulation facilitated redox homeostasis and cell wall remodeling without inducing oxidative damage, as evidenced by the stable MDA levels

Furthermore, untargeted metabolomics provided novel insights into the molecular basis of this growth promotion. We observed metabolic changes consistent with a strategic reprogramming, potentially involving a diversion of carbon and nitrogen flux from the free amino acid pool toward the shikimate and phenylpropanoid pathways. This putative metabolic shift suggests that SH-SeNPs could function as a “dual-purpose” regulator, acting as both a nutrient source and a metabolic primer that prepares the plant for potential defense while sustaining vegetative growth.

However, it is crucial to emphasize the boundaries of this study. Since our experiments were restricted to the seedling stage under controlled laboratory conditions, caution should be exercised in extrapolating these vegetative growth benefits to reproductive outcomes. The current data do not guarantee that the observed “efficient defense–robust growth” balance will persist through grain filling or result in final yield increases. To fully validate the agronomic potential of SH-SeNPs, long-term field trials quantifying selenium translocation to mature grains and assessing performance under complex environmental variables are indispensable.

## 5. Materials and Methods

### 5.1. Experimental Materials and Reagents

Fruit bodies of *P. igniarius* were collected from the Changbai Mountain region, Jilin Province, China. The samples were washed, air-dried, and ground into powder prior to use. Sodium selenite (Na_2_SeO_3_), ascorbic acid (AsA), and other chemical reagents of analytical grade were purchased from Sinopharm Chemical Reagent Co., Ltd(Shanghai, China). The rice cultivar used in this study was the japonica variety “Daohuaxiang No. 2”.

### 5.2. Extraction and Physicochemical Characterization of P. igniarius Polysaccharides

#### 5.2.1. Extraction of *P. igniarius* Polysaccharides

Polysaccharides were extracted from *P. igniarius* fruit bodies according to a previously reported method with minor modifications [51]. Briefly, dried *P. igniarius* powder was mixed with deionized water at a fixed solid-to-liquid ratio and subjected to ultrasound-assisted extraction at 80 °C, followed by water bath extraction. The extract was centrifuged to remove insoluble residues, concentrated under reduced pressure, and precipitated through the addition of absolute ethanol. The resulting precipitate was collected via centrifugation, redissolved in deionized water, and freeze-dried to obtain crude *P. igniarius* polysaccharides (SH).

#### 5.2.2. Composition and Molecular Weight Analysis of Polysaccharides

The crude polysaccharide (SH) yield was 0.93% (*w*/*w*). The total carbohydrate content was determined to be 82.30% using the phenol–sulfuric acid method [52], and the residual protein content was 2.17% as measured by the Coomassie Brilliant Blue assay [53]. Gel permeation chromatography (GPC) analysis revealed a number average molecular weight (*M_n_*) of 5.44 kDa with a polydispersity index of 1.71, indicating a relatively concentrated molecular weight distribution and, thus, suitability for nanoparticle stabilization.

### 5.3. Preparation and Optimization of Polysaccharide-Stabilized Selenium Nanoparticles

#### 5.3.1. Preparation of SH-SeNPs

The SH-SeNPs were synthesized following our previously optimized protocol with minor modifications using *P. igniarius* polysaccharides (0.5 g/L) [54]. Briefly, a sodium selenite solution at a predetermined concentration (up to 8000 mg/L) was added to the polysaccharide solution, followed by the slow dropwise addition of ascorbic acid as a reducing agent under magnetic stirring. The reaction temperature and time were carefully controlled, and the resulting suspension containing SH-SeNPs was obtained upon completion of the reaction [55]. High precursor concentrations were employed in this synthesis step to evaluate the maximum stabilizing capacity of the polysaccharides and to ensure high yield for potential large-scale agricultural applications. For subsequent foliar application experiments, the synthesized SH-SeNPs were diluted to working concentrations of 5, 45, and 85 mg/L.

#### 5.3.2. Single-Factor Experiments and Response Surface Optimization

The nanoparticle size was selected as the response variable to evaluate the effects of selenium concentration, selenite-to-reductant ratio, reaction time, and reaction temperature on the formation of SH-SeNPs. Based on the results of single-factor experiments, a Box–Behnken response surface methodology was employed to optimize key synthesis parameters, establish a mathematical model, and validate the optimal preparation conditions [56].

### 5.4. Structural and Physicochemical Characterization of SH-SeNPs

The morphology of the SH-SeNPs was observed using SEM and TEM. Particle size distribution and zeta potential were determined through dynamic light scattering (DLS) using a Zetasizer Nano ZS90 (Malvern, UK). The obtained SH-SeNPs exhibited a high absolute Zeta potential of −32.77 mV (at pH 7.0), which exceeds the threshold (±30 mV) typically required for electrostatic stabilization of nanosuspensions. This high surface charge, attributed to the capping effect of *P. igniarius* polysaccharides, prevented particle aggregation and maintained a uniform dispersion (bright red, transparent solution). To further evaluate colloidal stability, the optimized SH-SeNP suspension was stored at room temperature (25 °C) for 30 days, during which changes in visual appearance and particle size were monitored. UV–visible (UV–Vis) spectroscopy was used to analyze the characteristic absorption of selenium nanoparticles. Fourier transform infrared spectroscopy (FT-IR) was applied to investigate interactions between polysaccharides and selenium nanoparticles. Selenium valence states and elemental composition were analyzed via X-ray photoelectron spectroscopy (XPS), while the crystalline structure was characterized using X-ray diffraction (XRD) and Raman spectroscopy.

### 5.5. In Vitro Antioxidant Activity Assays

The in vitro antioxidant activities of SH-SeNPs were evaluated through DPPH, ABTS^+^, and superoxide anion radical scavenging assays [57,58]. Total antioxidant capacity was determined using the ferric reducing antioxidant power (FRAP) assay [59]. Non-selenized *P. igniarius* polysaccharides were used as controls, and antioxidant activities were compared at identical concentrations.

### 5.6. Rice Cultivation Conditions and Foliar Application Design

Rice seeds were surface-sterilized and germinated in the dark at 25 °C. Uniform seedlings were transferred to plastic containers (26.2 × 18.2 × 8 cm) containing 1.9 L of Kimura B nutrient solution and grown in a growth chamber with a 14 h light (28 °C)/10 h dark (20 °C) photoperiod at a relative humidity of 75%.

The experiment followed a randomized complete block design (RCBD) with three independent biological replicates for each treatment. Seedlings were divided into four groups: CK (water), SH (polysaccharide control), SeNPs (bare nanoparticles), and SH-SeNPs. Based on a preliminary screening, three selenium concentrations were selected: Low (5 mg/L), medium (45 mg/L), and high (85 mg/L). The 45 mg/L concentration was specifically chosen as a target dosage to achieve selenium levels compliant with the Chinese national standard for Se-enriched paddy (GB/T 22499—2008) [60]. Foliar sprays were applied twice a week for three consecutive weeks during the seedling stage. Each treatment consisted of three independent biological replicates, with 10 plants per replicate. while control plants received an equal volume of deionized water. For morphological measurements, 10 plants were randomly selected from each biological replicate to ensure statistical representativeness. Treatments were continued for three weeks, after which samples were collected for subsequent analyses.

### 5.7. Measurement of Growth Parameters and Physiological Indices

Plant height, root length, and fresh and dry weights of shoots and roots were measured. Chlorophyll content was determined spectrophotometrically [61]. Malondialdehyde (MDA) content [62] and the activities of superoxide dismutase (SOD) [63], peroxidase (POD) [64], and catalase (CAT) [65] were measured using commercial assay kits, according to the manufacturers’ instructions. Plant samples were harvested and separated into roots, stems, and leaves for selenium distribution determination. Tissue samples were dried at 70 °C, digested with HNO_3_-HClO_4_, and total selenium accumulation was quantified using atomic fluorescence spectrometry (AFS).

### 5.8. Metabolomic Analysis

#### 5.8.1. Sample Preparation and Metabolite Detection

Rice leaf samples from different treatments were harvested, immediately frozen in liquid nitrogen, and stored at low temperature. Metabolites were extracted and analyzed using ultra-high-performance liquid chromatography coupled with high-resolution mass spectrometry (UHPLC–HRMS). Data were acquired in both positive and negative ionization modes for untargeted metabolomic profiling [66].

#### 5.8.2. Quality Control and Data Processing

To ensure data reliability, quality control (QC) samples were prepared by pooling equal aliquots (10 μL) of all experimental samples. These QC samples were injected at the beginning of the sequence to equilibrate the system and subsequently injected after every 10 analytical samples to monitor instrument stability. Raw data were processed using Compound Discoverer 3.1. Prior to multivariate statistical analysis, data were standardized (Z-score transformation) and centered (mean centering) to minimize inter-sample variation and correct for heteroscedasticity. Differential metabolites were screened based on a Variable Importance in Projection (VIP) score > 1 and a *p*-value < 0.05 (Student’s *t*-test).

### 5.9. Statistical Analysis

All experiments were performed with at least three biological replicates, and the results are expressed as mean ± standard deviation. Statistical analyses were conducted using the SPSS software V30.0. Differences among treatments were evaluated via one-way analysis of variance (ANOVA), followed by Duncan’s multiple range test (DMRT) for post hoc multiple comparisons. Statistical significance was set at *p* < 0.05.

## Figures and Tables

**Table 1:** Box–Behnken experimental design and corresponding results for SH-SeNPs synthesis.

Run	Selenium Concentration (mg/L)	Selenite-to-Reductant Ratio (%)	Temperature (°C)	Particle Size (nm)
1	8000	33	60	179.7
2	6000	25	60	103.5
3	6000	25	60	102
4	6000	20	40	265.2
5	6000	25	60	101.2
6	6000	25	60	98.9
7	8000	25	80	245.1
8	8000	25	40	257.7
9	6000	33	80	193.3
10	4000	33	60	151.1
11	8000	20	60	299.8
12	6000	25	60	110
13	6000	20	80	241.9
14	4000	25	40	138.6
15	4000	25	80	166.8
16	4000	20	60	155.1
17	6000	33	40	137.6

**Table 2:** Analysis of variance (ANOVA) for the quadratic response surface model.

Source of Variation	Sum of Squares	df	Mean Square	*F Value*	*p Value*	Significance
Model	69,503.9	9	7722.66	131.64	<0.0001	**
A—Selenium concentration	14,416.16	1	14,416.16	245.73	<0.0001	**
B—Selenite-to-reductant ratio	11,272.51	1	11,272.51	192.15	<0.0001	**
C—Temperature	533.85	1	533.85	9.1	0.0195	**
AB	3441.52	1	3441.52	58.66	0.0001	**
AC	416.16	1	416.16	7.09	0.0323	**
BC	1595.28	1	1595.28	27.19	0.0012	**
A^2^	7759.03	1	7759.03	132.26	<0.0001	**
B^2^	14,276.57	1	14,276.57	243.35	<0.0001	**
C^2^	13,205.39	1	13,205.39	225.09	<0.0001	**
Residual	410.66	7	58.67			
Lack of fit	340.43	3	113.48	6.46	0.0516	
Pure error	70.23	4	17.56			
Total	69,914.56	16				

Note: ** indicates extremely significant differences (*p* < 0.01). *R^2^* = 0.9941, *R^2^* _Adj_ = 0.9866. A: selenium concentration; B: selenite-to-reductant ratio; C: Reaction temperature.

**Table 3:** Effects of different concentrations of SH, SeNPs, and SH-SeNPs on rice seed germination.

	CK	SH5	SH45	SH85	Se5	Se45	Se85	SS5	SS45	SS85
Shoot length (cm)	1.63 ± 0.27 b	1.89 ± 0.32 ab	2.05 ± 0.23 a	1.84 ± 0.30 ab	1.91 ± 0.21 ab	1.77 ± 0.19 ab	1.97 ± 0.37 a	1.98 ± 0.34 a	1.89 ± 0.35 ab	1.74 ± 0.31 ab
Root length (cm)	5.22 ± 0.78 c	5.97 ± 1.10 abc	5.71 ± 0.69 abc	5.66 ± 0.61 bc	5.50 ± 0.82 bc	5.44 ± 0.58 bc	6.52 ± 0.55 a	5.91 ± 0.50 abc	6.25 ± 0.89 ab	5.81 ± 0.82 abc

Note: Data are presented as mean ± standard deviation (n = 3). Different lowercase letters indicate significant differences among treatments (*p* < 0.05). CK: control; SH: *P. igniarius* polysaccharides; Se: bare SeNPs; SS: SH-SeNPs. Numbers following the abbreviations indicate concentrations in mg/L (e.g., SS45 = 45 mg/L SH-SeNPs).

## Data Availability

The data that support the findings of this study are available from the corresponding author upon reasonable request.
